# Purification and Cryo-Electron Microscopy Analysis of Bacterial Appendages

**DOI:** 10.21769/BioProtoc.5032

**Published:** 2024-07-20

**Authors:** Juan C. Sanchez, Joseph K. Baumgardt, Elizabeth R. Wright

**Affiliations:** 1 Department of Biochemistry, University of Wisconsin-Madison, Madison, WI, USA; 2 Biophysics Graduate Program, University of Wisconsin-Madison, Madison, WI, USA; 3 Biotechnology Training Program, University of Wisconsin-Madison, Madison, WI, USA; 4 DOE Great Lakes Bioenergy Research Center, University of Wisconsin, Madison, WI, USA; 5 Cryo-Electron Microscopy Research Center, UW-Madison, Madison, WI, USA; 6 Midwest Center for Cryo-Electron Tomography, Department of Biochemistry, University of Wisconsin, Madison, WI, USA; 7 Morgridge Institute for Research, UW-Madison, Madison, WI, USA

**Keywords:** Cryo-EM, Helical reconstruction, Bacterial flagellum, Polymers, Purification, Vitrification

## Abstract

A number of extracellular helical protein polymers are crucial for supporting bacterial motility. The bacterial flagellum is a polymeric appendage used to support cellular motility. Historically, structural studies of flagellar and other filaments were limited to those present as or locked into straightened states. Here, we present a robust workflow that produces biologically relevant high-resolution cryo-electron microscopy (cryo-EM) structures of bacterial flagellar filaments. We highlight how a simple purification method, centered around several centrifugation steps, exploits the process of filament ejection in *Caulobacter crescentus* and results in isolated filaments amenable to transmission electron microscopy (TEM) studies. The quality of the sample is validated by SDS-PAGE and negative stain TEM analysis before a sample is vitrified for cryogenic electron microscopy (cryo-EM) data collection. We provide a detailed protocol for reconstructing either straight or curved flagellar filaments by cryo-EM helical reconstruction methods, followed by an overview of model building and validation. In our hands, this workflow resulted in several flagellar structures below 3 Å resolution, with one data set reaching a global resolution of 2.1 Å. The application of this workflow supports structure-function studies to better understand the molecular interactions that regulate filament architecture in biologically relevant states. Future work will not only examine interactions that regulate bacterial flagellar and other filament organization but also provide a foundation for developing new helical biopolymers for biotech applications.

Key features

• Rapid high-quality purification of bacterial flagella via simple bacterial culturing, centrifugation, and resuspension methods.

• High-throughput cryo-EM data collection of filamentous objects.

• Use of cryoSPARC implementations of helical reconstruction algorithms to generate high-resolution 3D structures of bacterial flagella or other helical polymers.

## Graphical overview


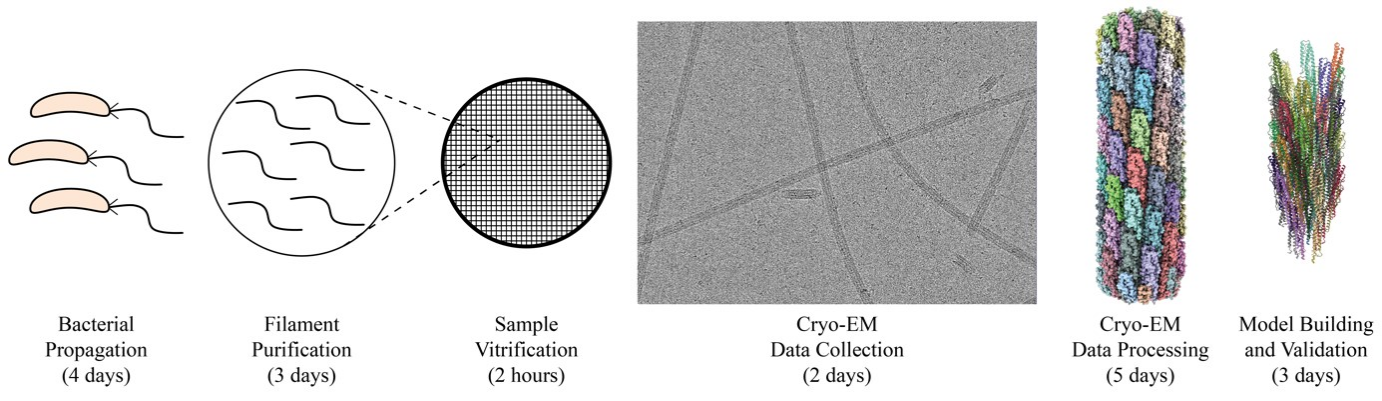


**Graphical overview of flagellar filament purification and cryo-EM imaging and analysis.** Following bacterial propagation of *C. crescentus*, cells are pelleted, and a series of centrifugation steps are used to collect ejected flagellar filaments. The filament quality is assessed before the sample is vitrified for cryo-EM data collection. Cryo-EM data is processed generating an electron potential map that is used for building an atomic model of the filament.

## Background

**Figure 1.**
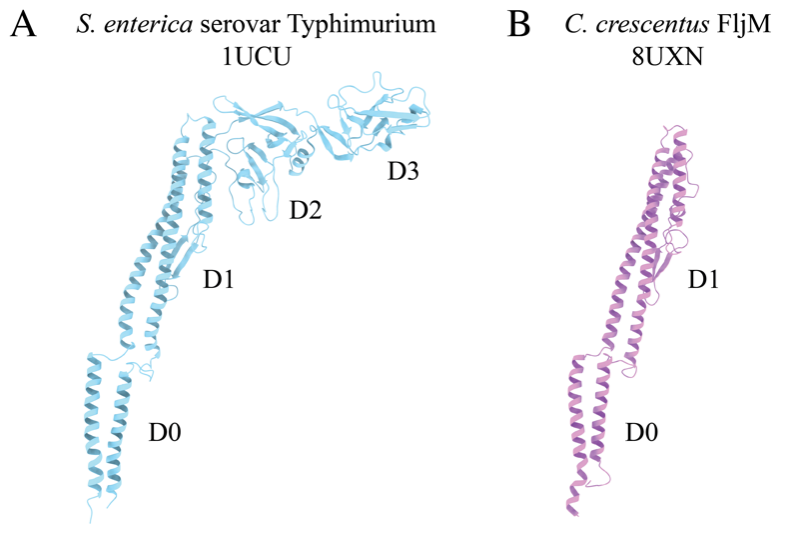
Comparison of bacterial flagellin models. A. Canonical flagellin model from *Salmonella enterica* serovar Typhimurium with D0, D1, D2, and D3 domains (PDB: 1UCU) [9]. B. *Caulobacter crescentus* FljM flagellin model with D0 and D1 domains, and lacking D2 and D3 domains (PDB: 8UXN) [21].

The flagellum is an appendage that supports motility in many bacterial species. The flagellum, an extracellular helical propeller, provides cells with a means to navigate through viscous environments. At the cell membrane, an ion-powered motor complex generates torque to rotate the helical filament. Although critical for cellular function, flagellum synthesis is an energetically expensive process that requires the regulated expression of over 60 genes to form a fully functional flagellum. These genes encode both structural and regulatory proteins [1,2]. Bacterial flagella are complex nanomachines, which have evolved to support the lifestyles and ecological niches of the associated species. It has been determined that ~45% of bacterial species possess multiple flagellin proteins, the structural proteins that comprise the flagellar filament [3,4]. In some species, only a single flagellin is synthesized and incorporated into the nascent filament due to phase variation, while others generate filaments comprised of multiple different flagellins [5–8]. Previously, studying the structure of these flexible helical polymers would require the introduction of point mutations to lock the flagellar filament into straightened forms [9,10]. Through these studies, it was determined that the flagellum was organized into 11 protofilaments made up of many flagellin monomers, each of which contains four domains, denoted D0, D1, D2, and D3 (Figure 1A). However, these studies were limited because little information could be gleaned about biologically relevant molecular interactions that impact flagellin subunit packing and filament architecture, i.e., without the imposed straightening mutations.

Our studies focus on the dimorphic bacterium, *Caulobacter crescentus.* The *C. crescentus* genome contains six flagellin genes that are expressed and incorporated into the wild-type filament [3,5]. Our structural analyses have shown that *C. crescentus* flagellins lack the D2 and D3 domains (Figure 1B) [11]. Interestingly, as *C. crescentus* undergoes cell division, a cell will eject the polar flagellar filament to allow for the synthesis of a polar stalk. The filament purification method presented here concentrates ejected filaments through a series of centrifugation and washing steps. The process of filament ejection can be artificially stimulated in other species when cells exhaust nutrients and enter the stationary phase [12,13]. Incorporating this step during bacterial propagation could allow our workflow to be applied to many other bacteria. The purified flagellar filaments were assessed by SDS-PAGE analysis and negative stain transmission electron microscopy (TEM) before cryo-EM studies. Although single-particle cryo-EM structure determination has exploded in the past decade, tools specific to iterative real-space helical reconstruction have lagged [14]. Fortunately, several new tools have become available that accelerate the reconstruction of helical polymers to high resolution [15–20]. Here, we demonstrate the application of those tools to determine the structures of both straight and curved helical polymers below 3.0 Å resolution. The protocol encompasses (1) methods to purify flagellar filaments from exhausted media, (2) detailed cryo-EM reconstruction workflows that use either symmetrized or asymmetrical reconstruction methods to resolve straight or highly curved filaments, and (3) an outline for model building and validation of large multimeric protein models based on the reconstructed maps. The protocols can be applied to studies of flagellar filament structures across multiple species. Additionally, the general cryo-EM workflow is not limited to flagellar filaments and can be applied to other helical biopolymers.

## Materials and reagents

**Biological materials**

1. *Caulobacter crescentus ΔfljJKLNO* (FljM only); *C. crescentus* strain with all flagellins deleted and the *fljM* flagellin gene introduced on a plasmid with kanamycin resistance (ERW2301) [21]
2. *Caulobacter crescentus ΔfljJLMNO* (FljK only); *C. crescentus* strain with genome deletions to all flagellins except for the *fljK* gene (TPA2353) [3]

**Reagents**

1. Peptone (Fisher Scientific, catalog number: BP1420-500)
2. Yeast extract (Fisher Scientific, catalog number: BP1422-500)
3. Bacto agar (BD Difco, catalog number: DF0140010)
4. Magnesium sulfate heptahydrate (Millipore Sigma, catalog number: MX0070)
5. Calcium chloride dihydrate (Sigma-Aldrich, catalog number: C3306-500G)
6. Kanamycin sulfate (Gibco, catalog number: 11-815-024)
7. Protease inhibitor cocktail (Millipore Sigma, catalog number: 539137)
8. Phosphate-buffered saline (1× PBS) (Corning, catalog number: 21-040-CV)
9. AnyKD Mini-PROTEIN TGX stain-free protein gels (Bio-Rad, catalog number: 4568126)
10. PageRuler Plus prestained protein ladder (Thermo Fisher, catalog number: 26619)
11. Coomassie Brilliant Blue (Sigma-Aldrich, catalog number: B7920-50G)
12. 2% uranyl acetate (UA) (EMS, catalog number: 22400-2)

**Solutions**

1. 1 M MgSO_4_ (see Recipes)
2. 1 M CaCl_2_ (see Recipes)
3. Peptone yeast extract (PYE) agar (see Recipes)
4. Peptone yeast extract (PYE) liquid media (see Recipes)
5. 1% Uranyl Acetate (see Recipes)

**Recipes**

1. **1 M MgSO_4_**
  **Table:** 

Reagent	Final concentration	Amount
Magnesium sulfate heptahydrate	1 M	61.62 g
H_2_O	n/a	250 mL
Total	n/a	250 mL
  - Dissolve the MgSO_4_ in water while stirring.
  - Filter the solution through a 0.22 µm filter sterile bottle top filter.
2. **1 M CaCl_2_**
  **Table:** 

Reagent	Final concentration	Amount
Calcium chloride dihydrate	1 M	36.75 g
H_2_O	n/a	250 mL
Total	n/a	250 mL
  - Dissolve the CaCl_2_ in water while stirring.
  - Filter the solution through a 0.22 µm filter sterile bottle top filter.
3. **Peptone yeast extract (PYE) agar**
  **Table:** 

Reagent	Final Concentration	Amount
Peptone	0.2% (w/v)	2 g
Yeast extract	0.1% (w/v)	1 g
Bacto agar	1.5% (w/v)	15 g
1 M MgSO_4_	1 mM	1 mL
1 M CaCl_2_	0.5 mM	0.5 mL
H_2_O	n/a	Fill up to 1 L
Total	n/a	1 L
  - Combine peptone, yeast, Bacto agar, and H_2_O in a flask and autoclave at 121 °C, 15 PSI for at least 20 min.
  - Allow the media to cool to ~55 °C before adding the salts.
*Note: For antibiotic-resistant strains, the media is supplemented with kanamycin at a concentration of 50 µg/mL.*
  - From 1 L of media, pour approximately 25 mL per plate into approximately 40 Petri plates (100 mm).
4. **Peptone yeast extract (PYE) liquid media**
  **Table:** 

Reagent	Final concentration	Amount
Peptone	0.2% (wt/v)	2 g
Yeast extract	0.1% (wt/v)	1 g
1 M MgSO_4_	1 mM	1 mL
1 M CaCl_2_	0.5 mM	0.5 mL
H_2_O	n/a	Fill up to 1 L
Total	n/a	1 L
  - Combine peptone, yeast, and H_2_O in a flask and autoclave at 121 °C, 15 PSI for at least 20 min.
  - Allow the media to cool to ~55 °C before adding the salts.
*Note: For antibiotic-resistant strains, the media was supplemented with kanamycin at a concentration of 50 µg/mL.*
5. **1% uranyl acetate (UA)**
  **Table:** 

Reagent	Final concentration	Amount
2% uranyl acetate	1% (v/v)	250 µL
H_2_O	n/a	250 µL
Total	n/a	500 µL
  Mix 1 part sterile water with 1 part 2% UA stain (EMS) and filter through a Spin-X centrifuge tube with a 0.22 µm filter (Costar).

**Laboratory supplies**

1. 100 mm × 15 mm Petri dishes (Fisher Scientific, catalog number: S33580A)
2. 250 mL vacuum filtration system (VWR, catalog number: 10040-464)
3. 1.7 mL centrifuge tubes (Denville, catalog number: C2170)
4. 0.22 µm Spin-X centrifuge tube filter (Costar, catalog number: 8160)
5. 200 mesh carbon film, copper grids (EMS, catalog number: CF200-CU)
6. Parafilm (Bemis, catalog number: PM996)
7. Whatman #1 filter paper (Whatman, catalog number: 1001-090)
8. Quantifoil R2/1 200 mesh, copper grids (Quantifoil Micro Tools GmbH, catalog number: Q210CR1)
9. Standard Vitrobot Filter Paper, Ø55/20 mm, Grade 595 (Ted Pella, catalog number: 47000-100)

## Equipment

1. NanoDrop spectrophotometer (Thermo Fisher, catalog number: ND2000)
2. Incubator with shaker (Labnet, catalog number: I-5311-DS)
3. 500 mL centrifuge bottles (Nalgene, catalog number: 3141-0500)
4. 70 mL polycarbonate bottle assembly with aluminum caps (Beckman Coulter, catalog number: 355622)
5. RC-5B Plus centrifuge (Sorvall, catalog number: SO-RC5B)
6. GS-3 rotor (Sorvall, catalog number: SO-GSA3)
7. Optima XL-80K ultracentrifuge (Beckman Coulter, catalog number: 8043-30-1211)
8. Type 45 Ti fixed angle titanium rotor (Beckman Coulter, catalog number: 339160)
9. Harvard Trip 1400/1500 Series balance (Ohaus, catalog number: 80000005)
10. 5424 R microcentrifuge (Eppendorf, catalog number: 05-400-005)
11. KS 260 basic shaker (IKA, catalog number: Z341835)
12. Plasma cleaner (Harrick Plasma Inc., catalog number: PDC-32G)
13. Static dissipator (Mettler Toledo, catalog number: UX-11337-99)
14. PTFE well plate (custom made; similar product: Kibron, catalog number: 6344)
15. Style N5 reverse pressure tweezers (Dumont, catalog number: 0202-N5-PS-1)
16. Talos L120C 120 kV transmission electron microscope (TEM) (Thermo Fisher Scientific), or equivalent
17. Cryo grid box (Sub-Angstrom, catalog number: SB)
18. Vitrobot Mark IV vitrification robot (Thermo Fisher Scientific)
19. Titan Krios G3i 300 kV transmission electron microscope (TEM) (Thermo Fisher Scientific)
20. K3-GIF direct electron detector with energy filter (Gatan Inc., AMETEK)
21. Supermicro server (KingStar) with two Xeon E5-2640 processors (Intel), 256 GB memory, and 4× 1080Ti GPU (NVIDIA), or equivalent setup.

## Software and datasets

1. CryoSPARC (https://cryosparc.com/)
2. IMOD (https://bio3d.colorado.edu/imod/)
3. AlphaFold2 (https://alphafold.ebi.ac.uk)
4. UCSF ChimeraX (https://www.cgl.ucsf.edu/chimerax/)
5. PHENIX (https://phenix-online.org/)
6. Coot (https://www2.mrc-lmb.cam.ac.uk/personal/pemsley/coot/)

## Procedure

- **Bacterial propagation**
  1. Using aseptic techniques, streak out the bacterial strains of interest onto the appropriate media to generate isolated colonies. Here, we isolated colonies of the *Caulobacter crescentus ΔfljJLMNO* (FljK only, no antibiotic resistance) and *ΔfljJKLNO* (FljM only, kanamycin resistance) strains. Cells were grown at 30 °C on PYE agar plates; for kanamycin-resistant strains, the media was supplemented with kanamycin at a concentration of 50 µg/mL.
*Note: These two strains only synthesize one out of the six flagellin structural proteins, resulting in mono-flagellin filaments. Additionally, the ΔfljJLMNO (FljK only, no antibiotic resistance) strain produces curved filaments, while the ΔfljJKLNO (FljM only, Kan^+^) strain produces straighter filaments.*
  2. Inoculate 5 mL of PYE liquid media with a single colony and incubate overnight at 30 °C shaking at 200 rpm.
  3. Using the overnight culture, inoculate 4 L of liquid media (split into three 1.3 L preps) and grow cells to exponential phase (OD_600_ = 0.6–0.8) at 30 °C with shaking at 200 rpm. In general, inoculating a 1.3 L culture with 300 µL of a *C. crescentus* overnight culture will take roughly 20–24 h to grow to exponential phase. We advise checking the OD after 18 h and then checking every 90 min until the desired OD is reached.
*Note: For example, for C. crescentus FljM filaments, 300 μL of overnight culture at an OD_600 _of 0.278 was used to inoculate the 1.3 L of PYE media, which resulted in 1.3 L of culture at an OD_600 _of 0.674 after 22 h and 30 min of incubation at 30 °C with shaking.*
- **Flagellar filament purification**
  1. Pellet cells at 10,000× *g* for 15 min using an RC-5B Plus centrifuge and GS-3 rotor with 500 mL centrifuge bottles or an equivalent setup.
  2. Decant the supernatant (containing ejected filaments) into clean flasks and discard the cell pellet. Ensure no visible cellular debris is collected with the supernatant. Repeat this step until all 3 L of bacterial culture are centrifuged. If needed, you can store the supernatant at 4 °C overnight or continue to the next step.
*Note: Although it is possible to store the supernatant beyond 24 h (i.e., over a weekend) before continuing to step B3, we have not studied the effects of prolonged supernatant storage on the integrity of the bacterial filaments.*
  3. Collect the flagellar filaments from the supernatant by centrifuging the supernatant at 35,000 rpm (~96,000× *g*) for 35 min at 4 °C using an Optima XL-80K ultracentrifuge and a Type 45 Ti fixed angle titanium rotor or a similar setup. For this procedure, we used 70 mL polycarbonate bottle assemblies with aluminum caps. Using a Harvard Trip balance, balance two tubes against each other and load them opposite of each other in the ultracentrifuge; repeat for additional tubes. Alternatively, a digital lab scale can be used here. Always ensure any tube loaded into the ultracentrifuge is filled to the top and balanced before centrifugation.
  4. Discard the supernatant and save the filament-containing pellet. Repeat the previous step with additional volumes of the supernatant until all the flagellar filaments are pelleted. Increase the size of the filament-containing pellet by adding fresh supernatant to the same tubes with the pellet after each spin. For efficiency, use two sets (six tubes per set) of tubes, ensuring one set is balanced and ready to load into the ultracentrifuge. This step takes approximately 6 h.
  5. Once all the supernatant is centrifuged and discarded, cover the filament-containing pellet with 10 mL of pre-chilled PBS buffer and incubate overnight at 4 °C with shaking at 100 rpm using a KS 260 basic shaker or an equivalent setup. This will help to resuspend the pellet without rigorous mechanical disruption to the filaments. While shaking, ensure the pellet is always suspended in the PBS buffer.
  6. Combine the resuspended pellets from previous steps. If you have two sets of 70 mL polycarbonate bottle assemblies with aluminum caps (12 tubes total), you should combine everything into two tubes containing ~60 mL of material each. Top off tubes with PBS and balance them.
  7. Centrifuge tubes at 13,500 rpm (~14,000× *g*) for 15 min at 4 °C using an Optima XL-80K ultracentrifuge and a Type 45 Ti fixed angle titanium rotor, or a similar setup, to pellet any large debris still present in the prep.
  8. Collect the supernatant by decanting the supernatant into a clean 70 mL polycarbonate bottle assembly and discard the cellular debris pellet.
  9. To collect flagellar filaments, centrifuge the supernatant at 35,000 rpm (~96,000× *g*) for 35 min at 4 °C using an Optima XL-80K ultracentrifuge and a Type 45 Ti fixed angle titanium rotor, or a similar setup.
  10. Discard the supernatant and save the filament pellet.
  11. Cover the pellet with 500 μL of PBS buffer and incubate overnight at 4 °C with shaking at 200 rpm using a KS 260 basic shaker or an equivalent setup. Arrange the tubes so the pellet is always suspended in the PBS buffer.
  12. Transfer the filament suspension to a clean 1.7 mL centrifuge tube. Cool the 5424 R microcentrifuge to 4 °C and centrifuge the tubes at 17,500× *g* for 15 min. Repeat once.
  13. Collect the supernatant in a clean 1.7 mL centrifuge tube and centrifuge at maximum speed (~21,000× *g*) for 2 h using the 5424 R microcentrifuge. Increase the temperature to 6 °C to prevent excessive ice buildup in the centrifuge.
  14. Discard the supernatant. Gently and quickly wash the pellet with 1 mL of PBS buffer and repeat once. Specifically, add 1 mL of PBS buffer to the side of the tube above the filament pellet; then, remove the PBS buffer without disturbing the pellet.
  15. Add 50 μL of pre-chilled PBS buffer and store the sample overnight at 4 °C under static conditions.
*Note: The purified sample may be stored for up to four weeks at 4 °C before loss of filament integrity. However, we encourage using the sample for the remaining experiments within two weeks of preparation, if possible. We have not characterized the effects of freeze/thaw cycles on sample integrity.*
  16. Run SDS-PAGE and stain with Coomassie Brilliant Blue stain or other staining reagent such as silver stain to assess the quality of the purification. Using an AnyKD Mini-PROTEIN TGX stain-free protein gel, we observed a band at ~25 kDa indicative of flagellin monomers for FljM and FljK filaments (Figure 2A and 2B, respectively). Lane 1 is a 4 μL aliquot from step B11 and lane 2 is a 2 μL aliquot from step B15. These additional steps greatly increase the filament concentration in the final pellet, as seen in the differences between lanes 1 and lanes 2 (Figure 2). The difference in protein quantity between strains is attributed to differences in filament lengths (Figure 2A compared to Figure 2B). The FljM strain produces smaller filaments (~3 µm) as compared to the FljK strain (~5.5 µm). Additionally, since the purification yields intact flagellar filaments, we observed bands above 70 kDa, which are likely hook-associated proteins (Figure 2).
    **Figure 2.**
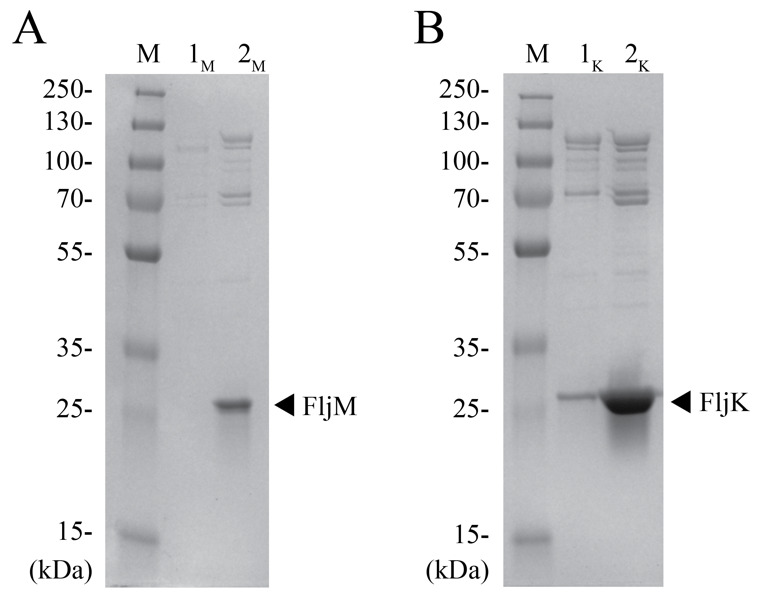
SDS-PAGE analysis of purified flagellar filaments. A. Lane M is an aliquot of the PageRuler Plus protein ladder, lane 1_M_ is an aliquot of the flagellar pellet after step B11, and lane 2_M_ is an aliquot of the final cell pellet for the *C. crescentus* FljM strain. B. Lane M is the protein ladder, lane 1_K_ is the flagellar pellet after step B11, and lane 2_K_ is the final cell pellet for the *C. crescentus* FljK strain. Arrows indicate the flagellin band at ~25 kDa for each strain.
- **Negative stain**
  Analysis by SDS-PAGE provides information about the concentration and purity of flagellin monomers. However, it does not inform us about the quality of the intact filaments. There is no biochemical assay to determine whether the filaments are intact. For this step, we use negative-stain TEM to assess the quality of the filaments and to determine a proper concentration of filaments to deposit on EM grids for vitrification.
  1. Glow-discharge 200 mesh carbon film, copper EM grids (EMS) with a plasma cleaner PDC-32G or an equivalent system (Figure 3, step 1).
  2. Line a PTFE well block (custom-made or Kibron) with a piece of parafilm and demagnetize with a static dissipater (Figure 3, step 2).
  3. Fill two wells of the PTFE well block with 50 µL of sterile, Nanopure water and two wells with 50 µL of 1% UA (Figure 3, step 3).
  4. Place 5 µL of the sample onto the EM grid and allow it to incubate for 1 min (Figure 3, step 4).
  5. Blot away the excess liquid by touching the blotting paper to the edge of the EM grid (Figure 3, step 5).
  6. Wash the EM grid in water by touching the face of the EM grid to a water droplet and blotting away excess liquid. Repeat once (Figure 3, step 6).
  7. Wash the EM grid once in a drop of 1% UA and blot away excess stain (Figure 3, step 7).
  8. Hold the EM grid face to a drop of UA stain for 15 s and blot away excess liquid (Figure 3, step 8).
  9. Allow the EM grid to dry for at least 5 min before storing for imaging (Figure 3, step 9).
  10. Repeat this process for additional sample dilutions to assess which concentration of filaments is best for cryo-EM analysis. We typically image a range of dilutions including 2×, 5×, and 10× sample dilutions in PBS buffer, as well as a concentrated sample.
  11. Image negative-stain EM grids on a Talos L120C 120 kV TEM (or equivalent) with a total electron dose of 20–30 e-/Å^2^ (Figure 4).
    **Figure 3.**
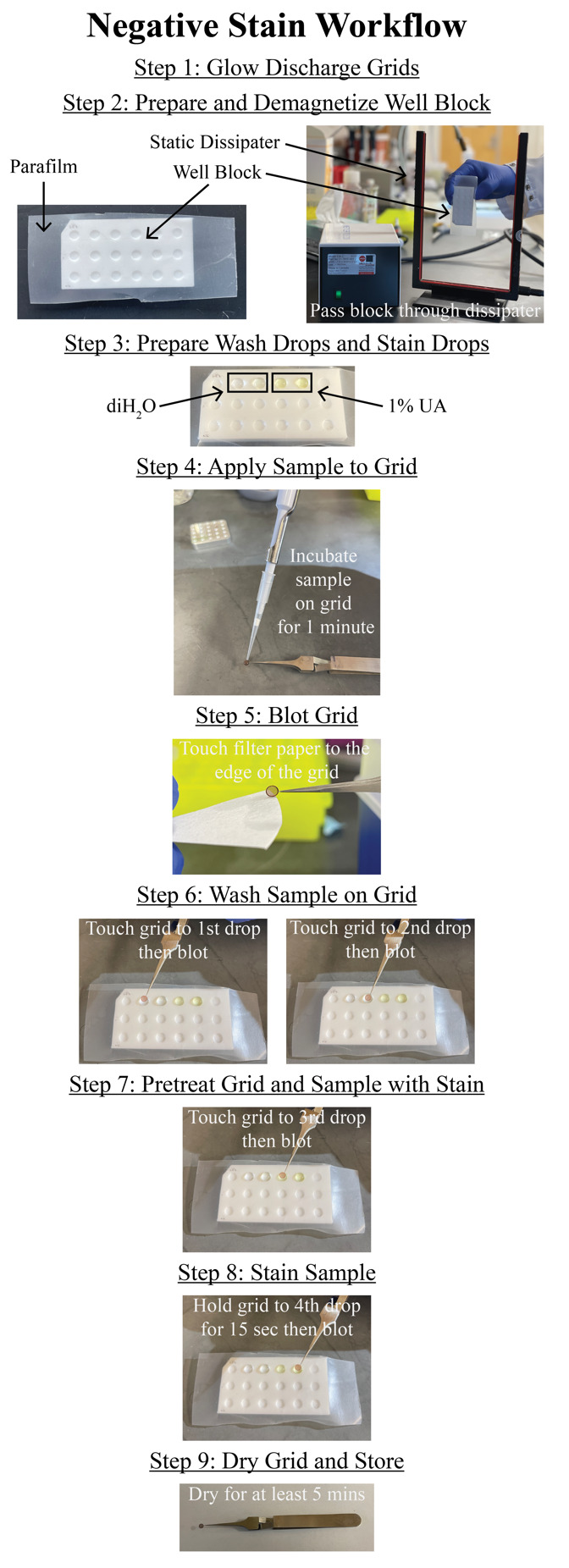
Negative-stain workflow. Step-by-step workflow for preparing negative stain grids of the purified *C. crescentus* flagella as detailed in the negative stain section. Numbering in the figure corresponds to numbering in Section C. All blotting steps are conducted as in step C5, by touching the edge of the grid to the filter paper. The procedure is repeated for replicate grids or different sample concentrations to be imaged by electron microscopy.
    **Figure 4.**
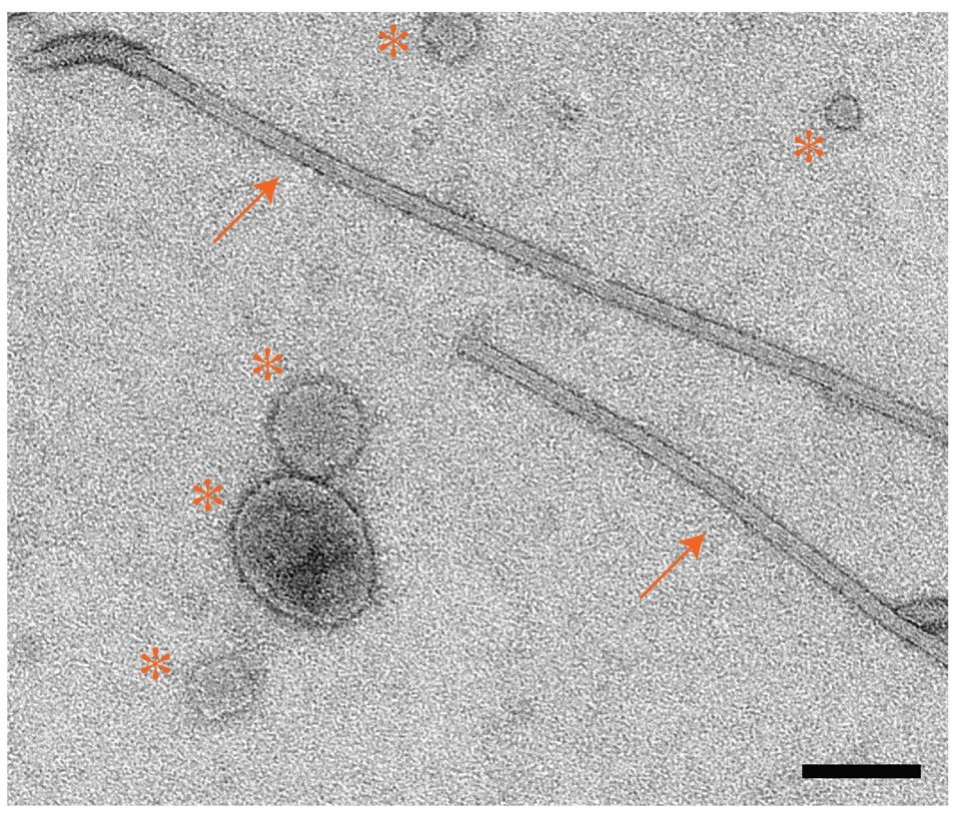
Negative-stain EM analysis of purified flagellar filaments. Micrograph of *C. crescentus* FljM flagellar filaments stained with 1% UA. Arrows indicate flagellar filaments; asterisks indicate extracellular vesicles that persist during the purification but do not inhibit cryo-EM data collection. Scale bar, 100 nm.
- **Sample vitrification**
  Sample vitrification using the Vitrobot Mark IV has become routine in the cryo-EM field. Below is a brief outline of the vitrification process with suggested blotting parameters that yield grids suitable for cryo-EM data collection.
  1. Add 60 mL of distilled water into the water reservoir.
  2. Turn on the Vitrobot Mark IV and set the relative humidity to 95% and the temperature to 20 °C.
  3. Attach standard Vitrobot filter paper to the blotting pads and allow time for the system to equilibrate (~15 min).
  4. Glow discharge R2/1 200 mesh, copper grids using a plasma cleaner or an equivalent system.
  5. Cool the Vitrobot foam dewar, ethane cup, and metal spider with LN_2_. Once the setup has cooled, condense the ethane in the ethane cup. Monitor ethane and LN_2_ levels throughout the entire vitrification process and top off accordingly.
  6. On the Vitrobot screen, set the drain time to 0.5 s and the wait time to 60 s. We freeze grids with blot times ranging from 2–4 s and a blot force of 0–6. It may be necessary to test a range of blotting conditions to determine what works best for your specific setup.
  7. Use the Vitrobot tweezers to pick up a grid, mount the tweezers onto the Vitrobot, and select *Continue* on the screen. Mount the foam dewar and follow the on-screen process.
  8. Apply 4 μL of your sample to the carbon side of the grid and select *continue* to proceed with the blotting conditions specified in step D6.
  9. Allow the system to plunge the specimen into the cryogen; then, transfer the vitrified grid to a labeled grid box for storage.
  10. Repeat steps D6–9 for the desired number of grids and ensure you test a range of blotting conditions and/or sample concentrations.
- **Cryo-EM data collection**
  Data collection parameters are specific to the microscope and detector used and should be adjusted according to the equipment available. Here, imaging was performed on a Titan Krios G3i FEG-TEM operated at 300 kV. Movies were collected on a Gatan K3 direct electron detector with a BioQuantum energy filter set to 20 eV. Dose-fractionated micrographs were collected in correlated-double sampling (CDS), counting mode spanning a relative defocus range of -0.5 to -2.5 µm with increments of 0.25 µm. A nominal magnification of 105,000× with a pixel size of 0.834 Å was used to acquire micrographs with a total dose of 45-55 e-/Å^2^ (1 e-/Å^2^/frame) (Figure 5). On average, ~250 movies were collected per hour using EPU/AFIS, acquiring three shots per hole and multiple holes per stage movement.
  **Figure 5.**
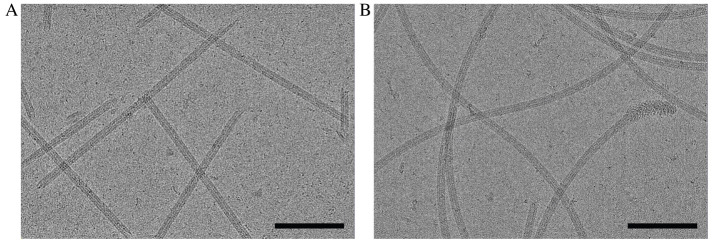
Representative cryo-EM micrographs of *C. crescentus* flagellar filaments. A. Motion-corrected micrograph of *C. crescentus* FljM flagellar filaments (straight). B. Motion-corrected micrograph of *C. crescentus* FljK flagellar filaments (curved). Scale bar, 100 nm.
- **Cryo-EM data processing of straight helical polymers (symmetrized refinement)**
  CryoSPARC was used to process *C. crescentus* flagellar filaments with an average diameter of 130 Å. *C. crescentus* flagellins range from 25 to 29 kDa and lack both D2 and D3 domains (Figure 1B). Only custom parameters are outlined below. If not specified, then default parameters were used. It is important to note that all datasets are unique, and one set of parameters may not be suitable for all needs; however, the steps below will greatly improve the user’s ability to differentiate between and analyze both straight and curved filaments. Further optimization may be needed on a case-by-case basis. The raw micrographs for both the straight FljM filaments and the curved FljK filaments are available on the EMPIAR database (entries EMPIAR-12122 and EMPIAR-12076, respectively) [22].
  As with any software program, it is best to complete the designed tutorial before processing unique datasets. We encourage new users to first complete cryoSPARC’s single-particle tutorial (https://guide.cryosparc.com/processing-data/get-started-with-cryosparc-introductory-tutorial) to become acquainted with the software [20].
  The preprocessing, particle picking, 2D classification, initial 3D refinement (Helical Refinement), and 3D variability analysis (steps F1–15 and G1–15) are the same for both the symmetrized (straight filaments) and asymmetrical (curved filaments) reconstruction workflows (Figure 6 and Figure 8). After completing these initial steps, it will be evident as to which path to continue. We have added a note after step F15 to help the user decide which workflow to use. For clarity, we have included all steps for both workflows, although the first 15 steps are the same, and we have provided a workflow diagram listing the steps performed for each reconstruction method (Figure 6 and Figure 8). Additionally, we provided movies with all the parameters for both reconstruction workflows to supplement the steps below (Video 1 and Video 2).
  **Figure 6.**
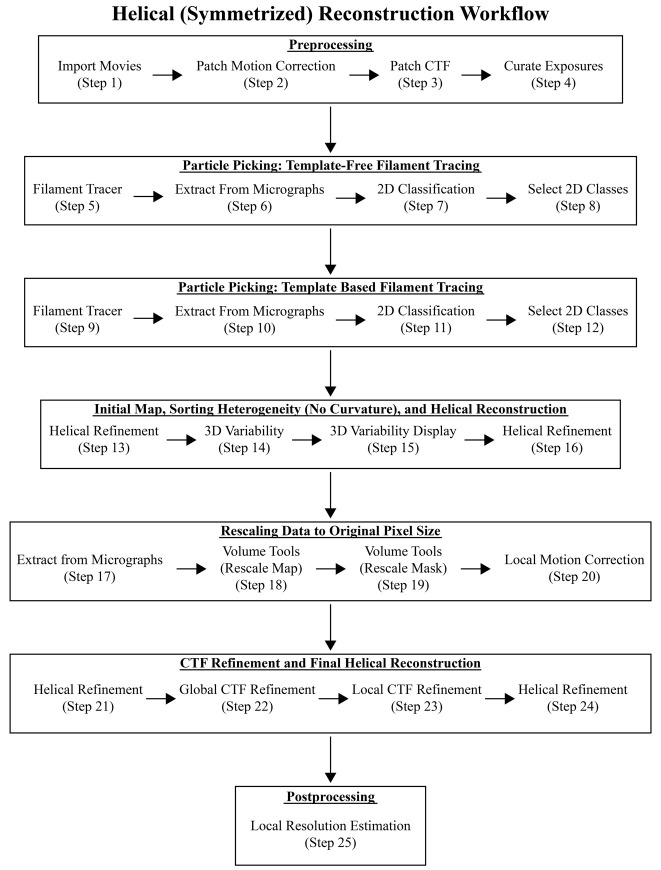
Workflow diagram of the helical (symmetrized) reconstruction method. The cryoSPARC jobs are provided here as a quick reference for processing data. These steps correspond to the steps in section F titled *Cryo-EM data processing of straight helical polymers (symmetrized refinement)* and to those in Video 1. Critical results are provided in Figure 7.
  **Video 1.**
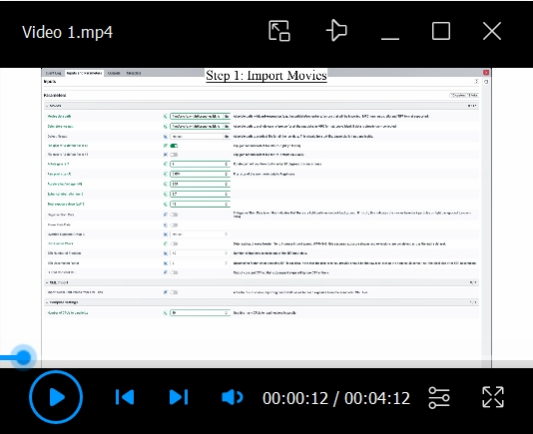
Cryo-EM data processing of straight helical polymers following symmetrized refinement procedures in cryoSPARC
  **Figure 7.**
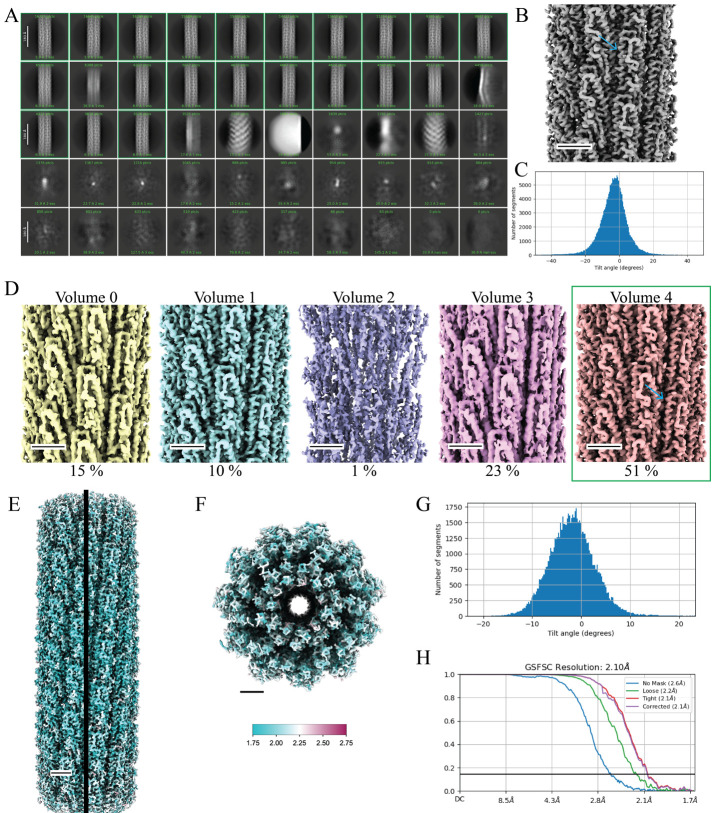
Cryo-EM data processing of straight helical polymers using symmetrized refinement yields high-resolution structures. A. Results of 2D classification after training the filament tracer neural net (step F11). Green boxes indicate classes that were selected for additional processing (step F12) (scale bar, 180 Å). B. 3D volume from the first helical refinement using a featureless cylinder as an initial reference (step F13). The blue arrow indicates α-helices that are present in the flagellin D1 domain. C. A Gaussian distribution of the particle tilt angles indicates proper symmetry was imposed in the 3D reconstruction, further verified by the high-resolution details present in the 3D volume (step F13). D. 3DVA helps to sort particle heterogeneity in the initial particle set. Volume 4 contains 51% of the particles and contains high-resolution features (blue arrow). Volume 1 also displays this quality but contains far fewer particles. The green box indicates Volume 4 was selected for further processing. E. Local resolution map of the final 3D volume along the helical axis F. Top view of the local resolution map of the final 3D volume. The key indicates a map range from 1.75 to 2.75 Å (step F25). G. The tilt angle plot from the final reconstruction displays a Gaussian distribution (step F24). H. The GS-FSC curve of the final map to a global resolution of 2.1 Å. The blue curve indicates the GS-FSC without masking, the green curve indicates the GS-FSC when a soft solvent mask extending 40 Å from the edge of the map is applied, the red curve indicates the GS-FSC when a tight mask extending only 12 Å from the edge of the map is applied, and the purple curve indicates the GS-FSC of the structure with a tight mask and correction by noise substitution. Scale bars for the 3D volumes are 25 Å.
  1. Import movies:
    We will start by assigning the proper path for *movies data* and *gain reference*. The *movies data* is the directory where raw micrographs are stored. Ensure the gain file is applied correctly by providing the proper flips and rotations. The gain parameters below are specific to our data collection. We first flip the gain file along the x-axis (left to right) and then rotate the file counterclockwise twice at 90°. You may need to seek advice from cryo-EM facility personnel where the data was collected for proper gain handling. To ensure micrograph dimensions are correct set *Skip Header Check* to *false*. Parallelize the job over multiple CPUs to speed up the processing.
*Flip gain ref & defect file in X: True*
*Rotate gain ref: 2*
*Raw pixel size (Å): 0.834*
*Accelerating voltage: 300*
*Spherical aberration (mm): 2.7*
*Total exposure dose (e-/Å^2^): 45*
*Skip header check: False*
*CPUs to parallelize: 10*
    We used 10 CPUs for a total run time of 55 min for 9,780 movies.
  2. Patch motion correction:
    Set the input to *Imported movies* from the **Import Movies** job (step F1). Run the default parameters and parallelize over multiple GPUs. We used four GPUs for a run time of 9 h and 17 min for 9,780 micrographs.
  3. Patch CTF:
    Set the input to *Micrographs* from the **Patch Motion Correction** job (step F2). Run the default parameters and parallelize over multiple GPUs. We used four GPUs for a total run time of 5 h for 9,780 micrographs.
  4. Curate exposures:
    Depending on the number of micrographs collected and the quality of the data, curating the dataset based on motion correction and CTF statistics may improve downstream results and decrease processing times. For this job, set the input to *Micrographs processed* from the **Patch CTF** job (step F3). Do not set auto thresholds. Instead, eliminate any outliers during the interactive session based on average intensity, average defocus, astigmatism, CTF fit resolution, defocus range, defocus tilt angle, relative ice thickness, total full-frame motion distance, and full-frame motion curvature. Here, we rejected 2,976 exposures and accepted 6,804 exposures for further processing.
  5. Filament tracer (template-free tracing):
    To improve automated picking, first generate a set of templates (2D classes) by running template-free tracing on a subset of your data (50–100 micrographs). We will then use these templates to retrain the **Filament Tracer** and apply the neural network to our entire dataset. For this job, set the input to *Exposures accepted* from the **Curate Exposures** job (step F4).
*Filament diameter: 130 Å*
*Separation distance between segments (diameters): 0.5*
*Minimum filament length (diameters): 3*
*Number of mics to process: 50*
*Number of mics to plot: 20*
*Minimum filament diameter: 120*
*Maximum filament diameter: 140*
    This job took 4 min to run. For initial estimates of filament diameter, open motion-corrected micrographs in IMOD or other visualization software to manually measure the filament diameter [23].
  6. Extract from micrographs:
    Here, we will set the box size for extracting particles. The box size should be set to at least 1.5× the filament diameter. Too small of a box size will result in no obvious curvature in the reconstruction of curved filaments. We use a box size spanning ~430 Å to observe the presence or lack of curvature in our reconstructions. Users may need to optimize this parameter during processing. Set the inputs to *All particles* and *Micrographs* from the **Filament Tracer** job (step F5). Parallelize over multiple GPUs to increase processing speeds.
*GPUs to parallelize: 2*
*Extraction box size: 512*
    This job took 1 min to extract 7,112 particles from 50 micrographs.
  7. 2D classification:
    Now, we will classify the particles to help remove suboptimal particles (non-flagella) before retraining the picking neural net. Set the input to *Particles extracted* from the **Extract From Micrographs** job (step F6).
*Number of 2D classes: 50*
*Maximum resolution (Å): 6*
*Initial classification uncertainty factor: 10*
*Align filament classes vertically: True*
*GPUs to parallelize: 2*
    Parallelizing over two GPUs resulted in a run time of 10 min for 7,112 particles.
  8. Select 2D classes:
    Set the inputs to *All particles* and *2D class averages* from the **2D Classification** job (step F7). The interactive interface will allow the user to manually select the best-looking classes. Of the 7,112 particles, 4,817 particles (68%) from 19 classes were selected and 2,295 particles (32%) from 31 classes were excluded.
  9. Filament tracer (with templates and a larger inter-box distance):
    We will use the best 2D classes from step F8 for template-based filament tracing. We will also increase the inter-box distance, i.e., the separation distance between particles, to avoid oversampling in our reconstruction. We aim for a separation of ~200 Å; however, in practice this value could be as small as one helical repeat or the unique asymmetrical unit in the helical polymer. Set the inputs to *Templates selected* from the **Select 2D Classes** job (step F8) and *Exposures accepted* from the **Curate Exposures** job (step F4).
*Filament diameter (Å): 130*
*Separation distance between segments (diameters): 1.6*
*Minimum filament length to consider (diameters): 3*
*Standard deviation of Gaussian blur (diameters): 0.2*
    This job resulted in 290,598 particles from 6,804 micrographs and took 10 h and 36 min to complete. It may be helpful to optimize these parameters on a small subset of micrographs prior to picking on the whole data set. To do so, provide an input to *setting number of micrographs to process* and *number of mics to plot.* For new datasets, we set both values to *50* to test picking parameters on only 50 micrographs and to view the picking quality in the log. Rerun the job with the optimized parameters on all the micrographs by removing the *setting number of micrographs to process* input value.
  10. Extract from micrographs:
    Binning particles during early steps improves particle alignment for 2D classification and 3D reconstruction jobs because this eliminates noise found at high-resolution frequencies that may lead to inaccuracies in particle alignments. Additionally, binning particles increase processing speeds during early iterations when particle sets are large due to the presence of suboptimal particles. Set the inputs to *All particles* and *Micrographs* from the latest **Filament Tracer** job (step F9).
*GPUs to parallelize: 2*
*Extraction box size (pix): 512*
*Fourier crop to box size (pix): 256*
    With the larger inter-box distance, we extracted 231,432 particles from 6,798 micrographs. This job took 1 h and 12 min to complete.
  11. 2D classification:
    This step allows for the removal of suboptimal particles in the data set. Additionally, because automated picking is not perfect, many times the hook structure or other high-contrast features may also be picked and need to be removed from the particle set. Set the input to *All particles* from the latest **Extract From Micrographs** job (step F10).
*Number of 2D classes: 50*
*Maximum resolution (Å): 6*
*Initial classification uncertainty factor: 10*
*Number of online-EM iterations: 40*
*GPUs to parallelize: 2*
    This job took 25 min to complete (Figure 7A).
  12. Select 2D classes:
    Set the inputs to *All particles* and *2D class averages* from the previous **2D Classification** job (step F11). From the interactive screen, select the classes with expected flagellar filament features. We selected 19 classes with 180,872 particles (78%) for further processing and excluded 31 classes with 50,560 particles (22%) (Figure 7A).
  13. Helical refinement (cylindrical initial model):
    For helical refinement, an initial estimate of helical symmetry parameters is necessary for the reconstruction of the correct volume. Initial estimates of helical symmetry parameters can be calculated from the averaged power spectrum of particles from one 2D class in a process called Fourier Bessel indexing. CryoSPARC has implemented an **Average Power Spectra** job that will compute the power spectrum for a selected 2D class, which can then be imported into programs such as HELIXPLORER, PyHI, and HI3D for indexing [15,18]. Alternatively, symmetry parameters from a homologous structure can be used as an initial estimate and refined during reconstruction. We have chosen the latter option for reconstructing flagellar filaments in this workflow. Set the input to *Particles selected* from the **Select 2D Classes** job (step 12).
*Helical twist estimate (°): 65.9*
*Helical rise estimate (Å): 4.8*
*Maximum out-of-plane tile angle (°): 45*
*Limit shifts along helical axis: True*
*Override outer filament diameter for search (Å): 140*
*Override inner filament diameter for search (Å): 10*
*Resolution to begin real-space symmetrization: 6*
*Resolution to begin local searches of helical symmetry: 4*
*Minimum helical twist to search over (°): 62.9*
*Maximum helical twist to search over (°):68.9*
*Twist grid size (number of grid points): 128*
*Minimum helical rise to search over (Å): 4.32*
*Maximum helical rise to search over (Å): 5.28*
*Rise grid size (number of grid points): 128*
*Initial lowpass resolution (Å): 20*
*Generate a cylindrical initial model: True*
*Filament outer diameter (Å): 140*
*Filament inner diameter (Å): 10*
    This job took 1 h and 7 min to complete. The gold standard Fourier shell correlation (GS-FSC_0.143_) reached the Nyquist limit of 3.4 Å for binned particles with a pixel size of 1.668 Å. Typically, for straight filaments, we observe secondary structures (α-helices) at this point in the reconstruction process (Figure 7B). Additionally, if the correct symmetry was imposed, we would expect to see a Gaussian distribution for the particle tilt angles (Figure 7C).
  14. 3D variability:
    We have found that results from 3D classification are suboptimal in cryoSPARC and instead prefer to run a **3D Variability** job to better sort heterogeneity within the particle set. Set the inputs to *All particles* and *Mask* from the **Helical Refinement** job (step F13). Ensure particles with 3D alignments are used for 3DVA, i.e., particles from any 3D refinement job. Additionally, memory issues are a concern when running 3DVA with large box sizes (> 500 pixels); thus, we will keep the particles binned during these steps.
*Number of modes to solve (eigenvectors): 3*
*Filter resolution (Å): 3.5*
    This job took 1 h and 20 min to complete.
  15. 3D variability display:
    Next, we will display 3DVA results based on component coordinates and group similar particles into clusters. The user can define the number of clusters to generate; here, we have chosen five clusters. Set the inputs to *All particles* and *3D Variability volumes* from the **3D Variability** job (step F14).
*Output mode: cluster*
*Number of frames/clusters: 5*
    This job took 2 min to complete. Use ChimeraX to analyze the results by first downloading the “*Volume series*” output file. Ideally, you will observe one class that has many particles and a structure with well-resolved secondary structures. Here, cluster 4 contained 77,284 particles and we could see α-helices with side chain densities (Figure 7D). The map from cluster 1 also contained a well-resolved volume but had only 16,339 particles. We chose cluster 4 particles for further processing; alternatively, we could combine both cluster 1 and cluster 4 particles for further processing.
*Note: This is where the processing workflows split between straight and curved filaments. As mentioned in step F13, if secondary structures are well resolved and the tilt angle plot displays a Gaussian distribution, then symmetrized (helical) reconstruction is best for your data set. However, if you are confident in the symmetry parameters, but the tilt angle plot displays a bimodal distribution, then there may be too much curvature in the particle set for adequate reconstruction via symmetrized (helical) refinement methods. The initial 3D volume (step F13) with helical symmetry imposed most likely has ill-defined secondary structures and few to no obvious side chain densities (i.e., “tubes” of density), and the GS-FSC may even reach the Nyquist limit for binned particles. Furthermore, 3D volumes from the 3DVA (steps F14 and 15) may appear curved along the helical axis as opposed to the symmetrized model from step F13. If these results are observed, then it is best to move on to the asymmetrical reconstruction workflow below (part G).*
  16. Helical refinement:
    We will rerun a refinement job after discarding suboptimal particles. Since we observed a Gaussian distribution for the tilt angles and a well-resolved map, we will continue to use the **Helical Refinement** jobs in this workflow (symmetrized reconstruction). We will use the refined helical parameters from the **Helical Refinement** job (step F13) as our initial estimates for helical symmetry. These values can be found at the end of the event log from step F13. Set the inputs to the volume and particles of your chosen cluster, in this case *Volume 4* and *Particles 4* from the **3D Variability Display** job (step F15).
*Helical twist estimate (°): 65.9*
*Helical rise estimate (Å): 4.732*
*Maximum out-of-plane tile angle (°): 10*
*Limit shifts along helical axis: True*
*Resolution to begin real-space symmetrization: 6*
*Resolution to begin local searches of helical symmetry: 4*
*Minimum helical twist to search over (°): 62.9*
*Maximum helical twist to search over (°):68.9*
*Twist grid size (number of grid points): 512*
*Minimum helical rise to search over (Å): 4.259*
*Maximum helical rise to search over (Å): 5.205*
*Rise grid size (number of grid points): 512*
    Initial *lowpass resolution (Å): 10*
*Mask (dynamic, static): static*
    This job took 23 min to complete. We observed a Gaussian distribution for tilt angles and the GS-FSC_0.143_ reached the Nyquist limit (3.4 Å) for this binned particle set. Symmetry parameters were refined resulting in a twist of 65.923° and a rise of 4.734 Å.
  17. Extract from micrographs:
    We curated our dataset to a point where resampling the particles to their original pixel size would lend to an increase in resolution and improve side-chain densities. Here, we will extract particles to the original pixel size without setting a value for the *Fourier crop to box size (pix)* parameter. Set the inputs to *All particles* from the previous **Helical Refinement** job (step F16) and to *Micrographs* from the **Extract From Micrographs** job (step F10).
*Extraction box size (pix): 512*
*Fourier crop to box size (pix): Not set*
*Recenter using aligned shifts: True*
    Using two GPUs, extracting 77,283 particles took 1 h and 42 min to complete.
  18. Volume tools (map resampling):
    In addition to resampling particles to the original pixel size, we must do the same for the map volume and mask generated in step F16. Set the input to *Refined volume* from the **Helical Refinement** job (step F16) and resample to the same box size as the final particles set (step F17).
*Resample to box size (pix): 512*
*Type of input volume: map*
    Type of output volume: map
    This job takes only seconds to run.
  19. Volume tools (mask resampling):
    Repeat the previous job for the mask volume. Set the input to *Mask* from the **Helical Refinement** job (step 16).
*Resample to box size (pix): 512*
*Type of input volume: mask*
*Type of output volume: mask*
    This job takes only seconds to run. At this point, we have resampled the particles, map, and mask to the original pixel size of 0.834 Å for additional processing.
  20. Local motion correction:
    Perform per particle motion correction to improve particle quality. Set the inputs to *Micrographs* and *Particles extracted* from the **Extract From Micrographs** job (step F17).
*Extraction box size (pix): 512*
    Using 2 GPUs this job took 2 h to complete.
  21. Helical Refinement:
    Set the inputs to *Particles extracted* from the **Local Motion Correction** job (step F20), *Volume* from the **Volumes Tools** (map resampling) job (step F18), and *Mask* from the **Volumes Tools** (mask resampling) job (step F19). We will use the refined helical parameters from the latest **Helical Refinement** job (step F16) as initial estimates of helical twist and rise. These values are stored at the end of the event log from step F16.
*Helical twist estimate (°): 65.923*
*Helical rise estimate (Å): 4.734*
*Maximum out-of-plane tile angle (°): 10*
*Limit shifts along helical axis: True*
*Resolution to begin real-space symmetrization: 6*
*Resolution to begin local searches of helical symmetry: 4*
*Minimum helical twist to search over (°): 62.923*
*Maximum helical twist to search over (°):68.923*
*Twist grid size (number of grid points): 512*
*Minimum helical rise to search over (Å): 4.261*
*Maximum helical rise to search over (Å): 5.207*
*Rise grid size (number of grid points): 512*
*Initial lowpass resolution (Å): 10*
*Mask (dynamic, static): static*
    This job took 2 h and 40 min to complete and resulted in a map with improved resolution (GS-FSC_0.143_ 2.23Å) and map quality.
  22. Global CTF refinement:
    To further improve the map, we will refine CTF parameters that were initially estimated at the start of our processing. Set the inputs to *All particles, Refined volume*, and *Mask* from the latest **Helical Refinement** job (step F21).
*Number of iterations: 5*
*Fit Tilt: True*
*Fit Trefoil: True*
*Fit Spherical Aberration: True*
*Fit Tetrafoil: True*
*Fit Anisotropic Magnification: True*
    This job took 13 min to complete.
  23. Local CTF refinement:
    Next, we will refine per particle defocus values. Set the inputs to *All particles* from the **Global CTF Refinement** job (step F22) and *Refined volume* and *Mask* from the latest **Helical Refinement** job (step F21). We will use default parameters for this job. This job took 5 min to complete.
  24. Helical refinement:
    Now that we have curated the particle set and refined both motion correction and CTF parameters, we are ready to perform the final refinement job. Set the inputs to *All particles* from the **Local CTF Refinement** job (step F23), and *Refined volume* and *Mask* from the latest **Helical Refinement** job (step 21). We will use the refined helical parameters from step F21 as initial estimates for helical symmetry parameters.
*Helical twist estimate (°): 65.923*
*Helical rise estimate (Å): 4.734*
*Maximum out-of-plane tile angle (°): 10*
*Limit shifts along helical axis: True*
*Resolution to begin real-space symmetrization: 6*
*Resolution to begin local searches of helical symmetry: 4*
*Minimum helical twist to search over (°): 62.923*
*Maximum helical twist to search over (°):68.923*
*Twist grid size (number of grid points): 512*
*Minimum helical rise to search over (Å): 4.261*
*Maximum helical rise to search over (Å): 5.207*
*Rise grid size (number of grid points): 512*
*Initial lowpass resolution (Å): 10*
*Mask (dynamic, static): static*
    The final refinement job took ~2 h and 50 min to complete and further increased the maps’ global resolution from 2.23 to 2.10 Å; the tilt angle plot displayed a Gaussian distribution (Figure 7G, 7H). Our final helical parameters were a twist of 65.923° and a rise of 4.732 Å. Multiple volumes are available for download under the *Refined volume* output. These include both half maps, a refined map, a sharpen map, a symmetrized map, and a sharpen symmetrized map. We find that building a model into the sharpen symmetrized map works best. However, the user may choose to run additional **Sharpening Tools** jobs and manually set a B-factor for sharpening either the map or the symmetrized map.
  25. Local resolution estimation:
    As a final post-processing step, we generate a local resolution volume to better understand the map quality using cryoSPARC’s BlocRes program. Set the inputs to *Refined volume* and *Mask* from the final **Helical Refinement** job (step F24).
*FSC threshold: 0.143*
*Use GPU or CPU for computation: GPU*
*Enable FSC weighting: True*
    Alternatively, the user can set *Enable MonoRes* to *True* to perform local resolution estimation with MonoRes instead of BlocRes. This job took 27 min to complete. The output file “*map_locres”*, along with the final map from step F24, can be opened in ChimeraX to generate a local resolution map (Figure 7E, 7F). In ChimeraX, go to **Volume > Surface Color**, set *Color surface* to the final map, set *by* to *volume data value*, and set *using map* to the local resolution map. Select *Options* to set the number of colors and the coloring palette desired. Select *Key* to add and edit parameters for the local resolution map key. For a detailed tutorial on coloring surface maps, see ChimeraX’s YouTube page (https://youtu.be/2EDZQs7lGNs?si=47v77qnzxLABJnBf).
- **Cryo-EM data processing of curved helical polymers (asymmetrical refinement)**
  Since some steps are similar for both workflows, we have noted steps where more details can be found in the previous section (symmetrized refinement).
  **Figure 8.**
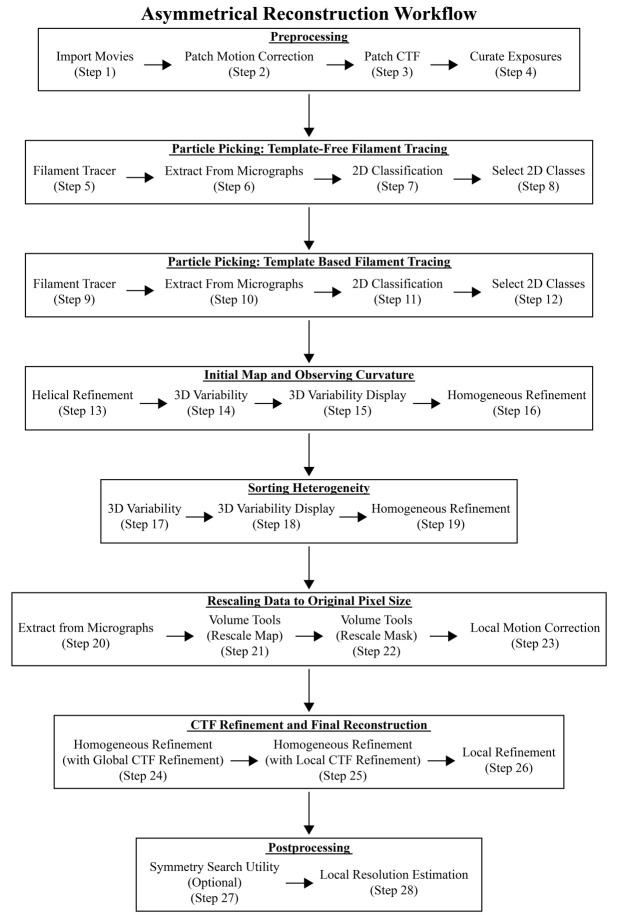
Workflow diagram of the asymmetrical reconstruction method. The cryoSPARC jobs are provided here as a quick reference when processing data. These steps correspond to the steps in section G titled *Cryo-EM data processing of curved helical polymers (asymmetrical refinement)* and to those in Video 2. Key results are detailed in Figure 9.
  **Video 2.**
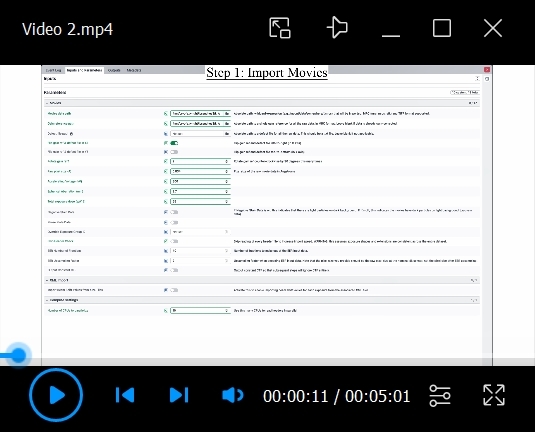
Cryo-EM data processing of curved helical polymers following asymmetrical refinement procedures in cryoSPARC
  1. Import movies:
    Assign the proper paths to *movies data* and *gain reference*. See step F1 on handling gain files.
*Flip gain ref & defect file in X: True*
*Rotate gain ref: 2*
*Raw pixel size (Å): 0.834*
*Accelerating voltage: 300*
*Spherical aberration (mm): 2.7*
*Total exposure dose (e-/Å^2^): 55*
*Skip header check: False*
*CPUs to parallelize: 10*
    We used 10 CPUs for a total run time of 1 h for 8,813 movies.
  2. Patch motion correction:
    Set the input to *Movies* from the **Import Movies** job (step G1). Running the default parameters on four GPUs resulted in a total run time of 10 h and 8 min for 8,813 micrographs.
  3. Patch CTF:
    Set the input to *Micrographs* from the **Patch Motion Correction** job (step G2). Running the default parameters on four GPUs resulted in a total run time of 4 h and 54 min for 8,813 micrographs.
  4. Curate exposures:
    Set the input to *Micrographs processed* from the **Patch CTF** job (step G3). Here, we manually rejected 2,835 exposures and accepted 5,978 exposures for further processing.
  5. Filament tracer (template-free tracing):
    Set the input to *Exposures accepted* from the **Curate Exposures** job (step G4). For more details, see step F5.
*Filament diameter: 130 Å*
*Separation distance between segments: 0.5*
*Minimum filament length: 3*
*Number of mics to process: 50*
*Number of mics to plot: 20*
*Minimum filament diameter: 120*
*Maximum filament diameter: 140*
    This job took 3 min to complete.
  6. Extract from micrographs:
    Set the inputs to *Micrographs* and *All particles* from the **Filament Tracer** job (step G5).
*GPUs to parallelize: 4*
    Extraction box size: 512
    This job took 1 min to extract 8,966 particles from 50 micrographs. See step F6 on box size selection.
  7. 2D classification:
    Set the input to *Particles extracted* from the **Extract From Micrographs** job (step G6).
*Number of 2D classes: 50*
*Maximum resolution (Å): 6*
*Initial classification uncertainty factor: 10*
*Align filament classes vertically: True*
*GPUs to parallelize: 4*
    Parallelizing over four GPUs resulted in a run time of 10 min for 8,966 particles.
  8. Select 2D classes:
    Set the inputs to *All particles* and *2D class averages* from the **2D Classification** job (step G7). Manually select the best-looking classes. Here, we selected 6,795 particles from 28 classes.
  9. Filament tracer (with templates and a larger inter-box distance):
    Set the inputs to *Templates selected* from the **Select 2D Classes** job (step G8) and *Exposures accepted* from the **Curate Exposures** job (step G4).
*Filament diameter (Å): 130*
*Separation distance between segments (diameters): 1.6*
*Minimum filament length to consider (diameters): 3*
*Standard deviation of Gaussian blur (diameters): 0.2*
    This job took 12 h and 21 min to complete and resulted in 372,299 particles from 5,978 micrographs. See step F9 for details on optimizing the filament tracer neural network.
  10. Extract from micrographs:
    Set the inputs to *All particles* and *Micrographs* from the **Filament Tracer** job (step G9). See step F10 for details on binning data. Here, we will resample the data from a pixel size of 0.834 Å to a 2× binned pixel size of 1.66 Å.
*GPUs to parallelize: 2*
*Extraction box size (pix): 512*
*Fourier crop to box size (pix): 256*
    This job took 1 h and 12 min to complete, resulting in 334,330 particles from 5,977 micrographs.
  11. 2D classification:
    Set the inputs to *All particles* from the previous **Extract From Micrographs** job (step G10).
*Number of 2D classes: 50*
*Maximum resolution (Å): 6*
*Initial classification uncertainty factor: 10*
*Align filament classes vertically: True*
*Remove duplicate particles: True*
*Minimum separation distance (for duplicate particles) (Å): 50*
*Number of online-EM iterations: 40*
*GPUs to parallelize: 2*
    This job took 28 min to complete (Figure 9A). We set *remove duplicate particles* to *true* as curved filaments will use **Homogeneous Refinement** jobs in later steps. If the sample is of a straight filament (symmetrized refinement), *remove duplicate particles* can be set to *false*, since cryoSPARC accounts for neighboring particles during the gold-standard split in **Helical Refinement** jobs.
  12. Select 2D classes:
    Set the inputs to *All particles* and *2D class averages* from the **2D Classification** job (step G11). We manually selected 31 classes with 310,135 particles (93%) and excluded 19 classes with 24,195 particles (7%) (Figure 9A).
  13. Helical refinement (cylindrical initial model):
    Set the input to *Particles selected* from the **Select 2D Classes** job (step G12). See step F13 for details on determining helical parameters.
*Helical twist estimate (°): 65.5*
*Helical rise estimate (Å): 4.8*
*Maximum out-of-plane tile angle (°): 45*
*Limit shifts along helical axis: True*
*Override outer filament diameter for search (Å): 140*
*Override inner filament diameter for search (Å): 10*
*Resolution to begin real-space symmetrization: 6*
*Resolution to begin local searches of helical symmetry: 4*
*Minimum helical twist to search over (°): 62.5*
*Maximum helical twist to search over (°):68.5*
*Twist grid size (number of grid points): 128*
*Minimum helical rise to search over (Å): 4.32*
*Maximum helical rise to search over (Å): 5.28*
*Rise grid size (number of grid points): 128*
*Initial lowpass resolution (Å): 20*
*Generate a cylindrical initial model: True*
*Filament outer diameter (Å): 140*
*Filament inner diameter (Å): 10*
    This job took 1 h and 32 min to complete and resulted in a map resolution (3.4 Å) that reached the Nyquist limit for the 2× binned data. For curved helical polymers, secondary structures are not as clear as straight filaments at this stage (Figure 9B). Additionally, we observe a bimodal tilt distribution in the **Helical Refinement** job (Figure 9C). These two data points suggest that an asymmetrical reconstruction may be necessary. We will run 3DVA to tease out whether curvature is inhibiting high-resolution reconstruction.
*Note: In some cases, it may be useful to repeat this step to improve map quality. Instead of using a featureless cylinder, use the resulting refined volume as in the initial model for a second round of helical refinement.*
  14. 3D variability:
    Set the inputs to *All particles* and *Mask* from the **Helical Refinement** job (step G13). See step F14 for details on 3DVA.
*Number of modes to solve (eigenvectors): 3*
*Filter resolution (Å): 3.5*
    This job took 2 h and 29 min to complete.
  15. 3D variability display:
    Next, set the inputs to *All particles* and *3D Variability volumes* from the **3D Variability** job (step G14).
*Output mode: cluster*
*Number of frames/clusters: 5*
    This job took 3 min to complete. Open the *Volume series* in ChimeraX to analyze the results of the 3DVA. Identify the best class, specifically looking at the quality of secondary structures. We determined *Volume 0* to be the best map with a slight curve to the filament along the helical axis.
  16. Homogeneous refinement:
    Next, we will rerun the 3D refinement, 3D variability, and 3D variability display jobs with the curved filament reconstruction from the **3D Variability Display** job (*Volume 0*, step G15) as the initial reference map. Set the inputs to *Particles 0, Particles 1, Particles 2, Particles 3, Particles 4*, and the best map (*Volume 0*) from the **3D Variability Display** job (step G15).
*Initial lowpass resolution (Å): 10*
    This job took 15 min to complete. We observe much higher map quality in this map as compared to the **Helical Refinement** job (step G13), although both resolve to a GS-FSC_0.143_ of 3.4 Å. Several subunits from the **Homogeneous Refinement** map display D1 domains with α-helices as opposed to a “tube” of volume observed in the **Helical Refinement** map (step G13) (Figure 9D). Bulky side chains and glycosylation sites are also better resolved.
  17. 3D variability:
    Although the quality of the map, with 2× binned particles, has greatly improved, it is important to further curate our particle set to further improve our results. Set the inputs to *All particles* and *Mask* from the **Homogeneous Refinement** job (step G16).
*Number of modes to solve (eigenvectors): 3*
*Filter resolution (Å): 3.5*
    This job took 2 h and 25 min to complete.
  18. 3D variability display:
    Next, set the inputs to *All particles* and *3D Variability volumes* from the **3D Variability** job (step G17).
*Output mode: cluster*
*Number of frames/clusters: 5*
    This job took 3 min to complete. The highest quality map was *Volume 0* with 45,319 particles. *Volume 1* through *Volume 4* lacked obvious side chain densities, had discontinuities to the carbon backbone density, or lacked clear α-helices (Figure 9E). In our experience, some datasets contain multiple “good” volumes; the user may decide to combine particles from the “good” classes for further processing or pick the single class with the highest particle count for further processing. We typically proceed with the latter option.
  19. Homogeneous refinement:
    Set the inputs to the best particle set(s) and volume(s) from the 3DVA. We set the inputs to *Particles 0* and *Volume 0* from the **3D Variability Display** job (step G18).
*Initial lowpass resolution (Å): 10*
    This job took 4 min to complete. We observe a slight improvement from the *Volume 0* map due to the automated sharpening as part of the **Homogeneous Refinement** job.
  20. Extract from micrographs:
    Now that we have curated our data to a final particle set, we will resample our pixel size to the original size (0.834 Å) for further processing. Set the inputs to *All particles* from the previous **Homogeneous Refinement** job (step G19) and to *Micrographs* from the **Extract From Micrographs** job (step G10).
*Extraction box size (pix): 512*
*Fourier crop to box size (pix): Not set*
*Recenter using aligned shifts: True*
    Using 1 GPU, extracting 44,978 particles took 3 h and 4 min to complete.
  21. Volume tools (map resampling):
    Next, we will resample the pixel size of the latest map to match the particles. Set the input as the *Refined volume* from the **Homogeneous Refinement** job (step G19) and resample the map to the same size as the particles in the previous step.
*Resample to box size (pix): 512*
*Type of input volume: map*
*Type of output volume: map*
    This job takes only seconds to run.
  22. Volume tools (mask resampling):
    Now resample the *Mask* from the **Homogeneous Refinement** job (step G19) to the original pixel size.
*Resample to box size (pix): 512*
*Type of input volume: mask*
*Type of output volume: mask*
    This job takes only seconds to run. Now, all the inputs for subsequent processing will have a pixel size of 0.834 Å and a box size of 512 pixels.
  23. Local motion correction:
    Now, we will perform per-particle motion correction to improve the quality of the particles. Set the inputs as the *Micrographs* and *Particles extracted* from the **Extract From Micrographs** job (step G20).
*Extraction box size (pix): 512*
    Using two GPUs, this job took 4 h and 9 min to complete.
  24. Homogeneous refinement (with global CTF refinement):
    Unlike helical refinement jobs, we can run global CTF refinements directly in the **Homogenous Refinement** job rather than running a stand-alone job. Set the inputs to *Particles extracted* from the **Local Motion Correction** job (step G23), *Volume* from the **Volume Tools** job (step G21), and *Mask* from the **Volume Tools** job (step G22).
*Initial lowpass resolution (Å): 10*
*Minimize over per-particle scale: True*
*Optimize per-group CTF params: True*
*Fit Tilt: True*
*Fit Trefoil: True*
*Fit Spherical Aberration: True*
*Fit Tetrafoil: True*
*Fit Anisotropic Mag: True*
    This job took 31 min to complete, resulting in a map with a GS-FSC_0.143_ of 3.05 Å.
*Note: If the resolution does not improve, run a stand-alone global CTF refinement job as in step F22 followed by a homogenous refinement job.*
  25. Homogeneous refinement (with local CTF refinement):
    Again, unlike helical refinement jobs, we can run local CTF refinement (defocus refinement) along with the **Homogenous Refinement** job. Set the inputs to *All Particles, Refined volume*, and *Mask* from the latest **Homogenous Refinement** job (step G24).
*Initial lowpass resolution (Å): 10*
*Optimize per-particle defocus: True*
    This job took 34 min to complete, and we observed a slight increase in the map’s global resolution to a GS-FSC_0.143_ of 2.82 Å.
*Note: If the resolution does not improve, run a stand-alone local CTF refinement as in step F23 followed by a homogenous refinement job.*
  26. Local refinement:
    As a final step, we perform a **Local Refinement** job to push our map quality a bit further. Set the inputs to *All Particles, Refined volume*, and *Mask* from the previous **Homogenous Refinement** job (step G25). Alternatively, the user may use ChimeraX to define a custom mask for this step. A step-by-step guide can be found on cryoSPARC’s website (https://guide.cryosparc.com/processing-data/tutorials-and-case-studies/mask-selection-and-generation-in-ucsf-chimera). Running the default parameters took 54 min and resulted in a slight increase in map quality and a GS-FSC_0.143_ of 2.72 Å (Figure 9H).
  27. Symmetry search utility:
    Although we are not applying helical symmetry to the final reconstruction, understanding the estimated helical arrangement of the subunits is useful information for comparison with the straight, symmetrized structure. This job takes a volume and provides the best local minima for helical parameters over a range of values defined by the user. The ranges for twist and rise will be unique for the helical polymer; see step F13 for details on estimating helical symmetry parameters. Set the inputs to the *Refined volume* and *Mask* from the **Local Refinement** job (step G26).
*Search over pitch/number of subunits, or rise/twist: rise*
*Search min and max over helical rise (Å): 4,5.5*
*Search min and max over helical twist (°): 55,75*
*Override outer filament diameter for search (Å): 200*
*Override inner filament diameter for search (Å): 10*
*Which map to search: map_sharp*
    This job took 2 min to complete and resulted in a table, found in the event log, that details the 20 best local minima of helical symmetry parameters. The values are arranged by mean squared error (MSE), a measurement of how close the experimental data is to the predicted model. A lower MSE suggests there is higher confidence in the estimated symmetry parameters. A twist of 65.51° and a rise of 4.812 Å best detail the arrangement of subunits in this flagellar filament, having the lowest MSE value of the local minima examined.
  28. Local resolution estimation:
    Finally, we will generate a local resolution map to detail the quality of our final reconstruction. Set the inputs to the *Refined volume* and *Mask* from the **Local Refinement** job (step G26).
*FSC threshold: 0.143*
*Use GPU or CPU for computation: GPU*
*Enable FSC weighting: True*
    This job took 54 min to complete (Figure 9F, 9G). See step F25 for further details on using ChimeraX to apply the local resolution plot to the final map.
    **Figure 9.**
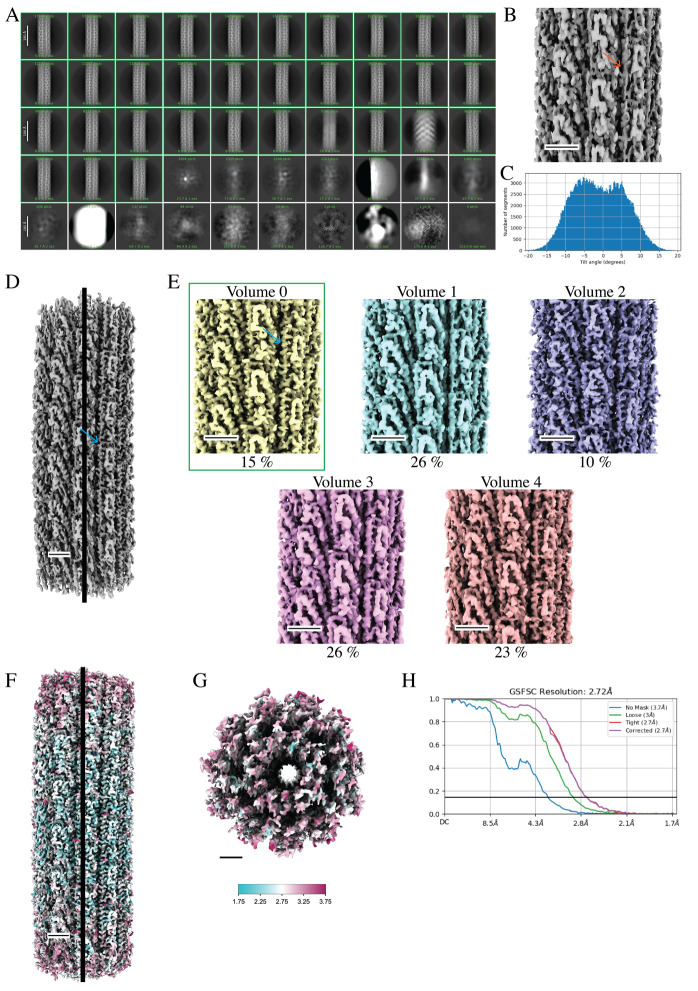
Cryo-EM data processing of curved helical polymers by asymmetrical reconstruction methods improves map quality. A. 2D classes from particles that were picked after training the filament tracer neural net (step G11). Green boxes highlight classes that were selected for further processing (step G12) (scale bar, 180 Å). B. Initial 3D volume with imposed helical symmetry results in the blurring of secondary structures. The red arrow indicates an expected α-helix that is blurred and more “tube-like” (step G13). C. Bimodal distribution of particle tilt angles suggests that the high curvature observed in the micrographs impedes high-resolution structure determination when imposing helical symmetry (step G13). D. Curved 3D volumes are observed after 3DVA. The best volume is used as a reference for one round of homogeneous refinement (no helical symmetry imposed) using all particles. This process results in a map with much higher quality and improved secondary structure features (blue arrow). Black line indicates the center of the 3D volume. E. A second round of 3DVA helps to sort heterogeneity within the particle set. Volume 0 has the best-resolved α-helices (blue arrow) and is selected (green box) for further processing. F. The selected particle set is used for additional rounds of homogeneous reconstruction, CTF refinement, and local motion correction, resulting in a final map with improved map quality displaying secondary structures and side-chain densities. The local resolution map shows that the highest resolved density is along the center of the helical axis. G. A top view of the filament shows lower resolution density at the end of the reconstruction. The key indicates a map range from 1.75 to 3.75 Å (step G26). H. The GS-FSC reports a global resolution of 2.72 Å. Scale bars for the 3D volumes are 25 Å.
- **Model building and validation**
  To accelerate model building, a predicted model or a previously solved structure can be used as an initial starting point. Alternatively, the user may manually build the protein chain using Coot. Here, we will outline how we use AlphaFold to generate a predicted model that is then manually and automatically refined into the experimental data using ChimeraX, Coot, and Phenix, in an iterative fashion (Figure 10) [24–27]. With the growing number of structure predictions already stored in the AlphaFold database, it is best to first search for your protein of interest in the AlphaFold database (https://alphafold.ebi.ac.uk). If your structure prediction is available, download the PDB file and move to step H3. If not, follow all the steps below.
  Additionally, there are many workflows that combine various software packages for model refinement. For less-experienced users, DiNunno et al. [28] provided a detailed protocol centered on modeling and validation.
  **Figure 10.**
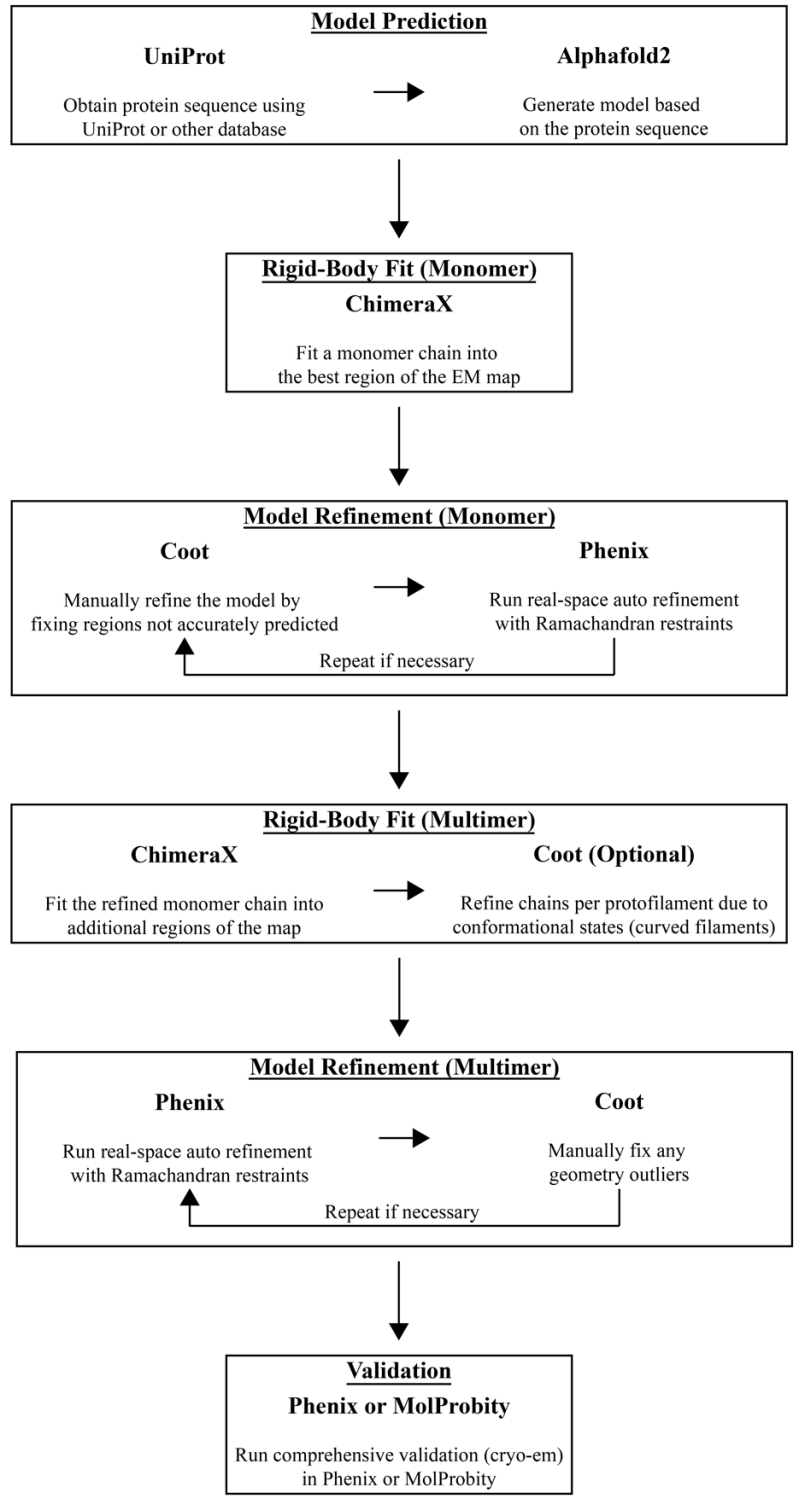
Model building and validation workflow using AlphaFold2, ChimeraX, Coot, and PHENIX
  1. Obtain the protein sequence using Uniprot or another database of choice.
  2. Go to the AlphaFold Colab notebook (https://colab.research.google.com/github/deepmind/alphafold/blob/main/notebooks/AlphaFold.ipynb), follow the steps for generating an initial prediction, and download the PDB file.
  3. Open the final cryo-EM map and the AlphaFold PDB file in ChimeraX and fit the model into the map using **Tools > Volume Data > Fit in Map**. First, you will need to manually align the model to the map followed by rigid-body fitting the model into the map using the **Fit in Map** tool. Save the PDB file ensuring you select *Save relative to model:* and choose the correct map from the drop-down menu. Your model is now aligned relative to your cryo-EM map.
  4. Open the aligned model and cryo-EM map in Coot. Click on the *Map* button (right-hand side) and then click *Estimate* to automatically estimate the weight for map fitting. Next, select **Refine > Chain Refine** to run an all-atom real space refinement on the model. Inspect the model to ensure the carbon backbone fits into the map density. The user can manually drag atoms into the corresponding density. Once satisfied, accept the refinement results.
  5. Run a validation analysis to identify outliers that need to be fixed. Select **Validate > Ramachandran Plot > ModelName.PDB**, followed by **Validate > Rotamer Analysis > ModelName.PDB**, and **Validate > Geometry analysis > ModelName.PDB**. Fix outliers on these plots by using the *Real Space Refine Zone* tool, *Auto Fit Rotamer* tool, or other model refinement tools on the right-hand panel. Once outliers are fixed, save the file coordinates.
  6. Open PHENIX to start a real-space refinement job. In the *Input/Output* tab, provide the proper map (MRC format) and model (PDB format) files. Also, provide a global resolution in the *Resolution* box. Under the *Refinement settings* tab, in addition to running the default parameters, select *Use secondary structure restraints.* Click on the *Rotamers* button and for *Fit* select *outlier_or_poormap* from the drop-down menu. For *Nproc*, define how many CPUs to run to speed up calculations; we typically set this to *4.* Click *Run* at the top of the window. Once the job is complete, inspect the *Validation* tab to review the results.
  7. Sometimes, it may be necessary to repeat step H4 and/or step H5 to improve atoms or residues that were flagged in the validation report. Repeat these steps as necessary until the validation statistics are satisfactory.
  8. We can now use the refined model to fit additional protein chains into the cryo-EM map by repeating step H3. In the symmetrized maps, we confidently built 44-mer models; in the asymmetrical maps, we built approximately 36 chains per map. Once all the chains are placed into the cryo-EM density map, we must combine the separate chain models into one multimeric model containing all the protein chains. From the ChimeraX command line, run “combine #1-44” where the integers after the “#” dictate the model IDs in ChimeraX. This command will combine 44 separate chains into one model and rename the chains, ensuring each one has a unique chain ID. Programs such as PHENIX require all chains to be in one model for refinement.
*Note: As an optional step, we can repeat steps H3, H4, and H5 for a chain from each protofilament to improve auto-refinement of the multimeric structure. This is useful for curved filaments where the D1 domain is in a different confirmation in each of the 11 protofilaments. After refining one chain per protofilament, ensure that you fit additional chains into the corresponding protofilament. We typically fit 3–4 chains per protofilament. Repeat for each protofilament and combine the many chains into one model as described above.*
  9. Run PHENIX’s real-space refinement as in step H5 ensuring the multimeric model is supplied in the *Input/Output* tab. Also, if the model was fit into a symmetrized map, you can select *NCS constraints* in the *Refinement Settings* tab to apply non-crystallographic symmetry during refinement. Review the validation results.
  10. These large flagellar filament models encompass tens of thousands of atoms and, although automatic refinement greatly improves the model, manual intervention is usually necessary. Use Coot to touch up any outliers that may appear in the PHENIX validation log. Save the model coordinates once you are satisfied with the model.
  11. In PHENIX, run the comprehensive validation (cryo-EM) tool. In the *Input/Output* tab, supply the model (PDB format), the final map (MRC format), and both half-maps (MRC format). Provide the map resolution and click *Run*. Once completed, review the model validation statistics.
  12. If there are any outliers, repeat step H9 and/or H10 until results are satisfactory.
  13. As an additional validation measure, submit the results to the PDB validation system (https://validate-rcsb-1.wwpdb.org).

## Data analysis

Data processing for any cryo-EM project is dependent on the sample, data collection instrumentation, and computational hardware and software. The protocol and workflow presented here were used to generate cryo-EM maps and atomic models for *C. crescentus* FljM and FljK flagellar filaments. Atomic models have been deposited in the Protein Data Bank under accessions 8UXN and 8UXJ for FljM and FljK, respectively. Cryo-EM maps have been deposited in the Electron Microscopy Data Bank under accessions EMD-42770 and EMD-42766 for FljM and FljK, respectively.

## Validation of protocol

This protocol or parts of it has been used and validated in the following research articles:

- Montemayor et al. [11]. Flagellar Structures from the Bacterium Caulobacter crescentus and Implications for Phage ϕ CbK Predation of Multiflagellin Bacteria. Journal of Bacteriology. https://doi.org/10.1128/jb.00399-20 (Figure 5, panel A).
- Sanchez et al. [21]. Atomic-level architecture of Caulobacter crescentus flagellar filaments provide evidence for multi-flagellin filament stabilization. bioRxiv. https://doi.org/10.1101/2023.07.10.548443 (Figure 1, panel A–I).

## General notes and troubleshooting

**Limitations**

This protocol is for the isolation and purification of flagella ejected from bacterial cells, cryo-EM imaging of the purified flagella, and single particle helical reconstruction of flagella using cryoSPARC software. We have tested the protocol on other filamentous polymers, including bacterial pili. However, we have not exhaustively tested the protocol on a large range of helical polymers because this may require additional optimization of steps associated with sample purification and/or cryo-EM data processing.

**Troubleshooting**

The procedures and workflows described in the protocol are starting points for individuals new to cryo-EM data collection and data analysis of filamentous targets that are straight or exhibit curvature. Samples are themselves variable and the protocol may require changes for optimal production and purification. These could include bacterial strain, growth medium, growth time, pH and salt concentrations of buffers, and types and timing of centrifugation and resuspension steps; each of these may need assessment and optimization to produce samples suitable for cryo-EM data collection. In addition, the hardware and software solutions for cryo-EM data collection and data processing may change over time, thus improving throughput and overall outcomes.

## References

[1] Jarrell K. F. (2009). Pili and flagella: current research and future trends. Caister Academic Press. Norfolk. ISBN: 9781904455486.

[2] Subramanian S. and Kearns D. B. (2019). Functional Regulators of Bacterial Flagella. Annu Rev Microbiol. 73: 225-246. 31136265 10.1146/annurev-micro-020518-115725 PMC7110939

[3] Faulds-Pain A., Birchall C., Aldridge C., Smith W. D., Grimaldi G., Nakamura S., Miyata T., Gray J., Li G., Tang J. X., .(2011). Flagellin redundancy in Caulobacter crescentus and its implications for flagellar filament assembly. J Bacteriol. 193(11): 2695-2707. 21441504 10.1128/JB.01172-10 PMC3133132

[4] Kanehisa M., Araki M., Goto S., Hattori M., Hirakawa M., Itoh M., Katayama T., Kawashima S., Okuda S., Tokimatsu T., .(2008). KEGG for linking genomes to life and the environment. Nucleic Acids Res. 36: D480-D484. 18077471 10.1093/nar/gkm882 PMC2238879

[5] Driks A., Bryan R., Shapiro L. and DeRosier D. J. (1989). The organization of the Caulobacter crescentus flagellar filament. J Mol Biol. 206(4): 627-636. 2738912 10.1016/0022-2836(89)90571-8

[6] Luo Y., Wang J., Gu Y. L., Zhang L. Q. and Wei H. L. (2023). Duplicated Flagellins in Pseudomonas Divergently Contribute to Motility and Plant Immune Elicitation. Microbiol Spectr. 11(1). Doi: 10.1128/spectrum.03621-22 PMC9927476 36629446

[7] Nuijten P. J., van Asten F. J., Gaastra W. and van der Zeijst B. A. (1990). Structural and functional analysis of two Campylobacter jejuni flagellin genes. J Biol Chem. 265(29): 17798-17804. 2211662

[8] Lederberg J. and Iino T. (1956). Phase Variation in Salmonella. Genetics. 41(5): 743-757. 17247660 10.1093/genetics/41.5.743 PMC1209814

[9] Yonekura K., Maki-Yonekura S. and Namba K. (2003). Complete atomic model of the bacterial flagellar filament by electron cryomicroscopy. Nature. 424(6949): 643-650. 12904785 10.1038/nature01830

[10] Wang F., Burrage A. M., Postel S., Clark R. E., Orlova A., Sundberg E. J., Kearns D. B. and Egelman E. H. (2017). A structural model of flagellar filament switching across multiple bacterial species. Nat Commun. 8(1): 1-13. 29038601 10.1038/s41467-017-01075-5 PMC5643327

[11] Montemayor E. J., Ploscariu N. T., Sanchez J. C., Parrell D., Dillard R. S., Shebelut C. W., Ke Z., Guerrero-Ferreira R. C. and Wright E. R. (2021). Flagellar Structures from the Bacterium Caulobacter crescentus and Implications for Phage varphi CbK Predation of Multiflagellin Bacteria. J Bacteriol. 203(5). Doi: 10.1128/jb.00399-20. PMC7890551 33288623

[12] Ferreira J. L., Gao F. Z., Rossmann F. M., Nans A., Brenzinger S., Hosseini R., Wilson A., Briegel A., Thormann K. M., Rosenthal P. B., .(2019). γ-proteobacteria eject their polar flagella under nutrient depletion, retaining flagellar motor relic structures. Plos Biol. 17(3): e3000165. 30889173 10.1371/journal.pbio.3000165 PMC6424402

[13] Zhuang X. Y. and Lo C. J. (2020). Construction and Loss of Bacterial Flagellar Filaments. Biomolecules. 10(11). 10.3390/biom10111528 PMC7696725 33182435

[14] Egelman E. H. and Wang F. (2021). Cryo-EM is a powerful tool, but helical applications can have pitfalls. Soft Matter. 17(12): 3291-3293. 33729271 10.1039/d1sm00282a PMC8086904

[15] Zhang X. (2022). Python-based Helix Indexer: A graphical user interface program for finding symmetry of helical assembly through Fourier-Bessel indexing of electron microscopic data. Protein Sci. 31(1): 107-117. 34529294 10.1002/pro.4186 PMC8740834

[16] Bepler T., Morin A., Rapp M., Brasch J., Shapiro L., Noble A. J. and Berger B. (2019). Positive-unlabeled convolutional neural networks for particle picking in cryo-electron micrographs. Nat Methods. 16(11): 1153 1160 1160.. 31591578 10.1038/s41592-019-0575-8 PMC6858545

[17] Wagner T., Merino F., Stabrin M., Moriya T., Antoni C., Apelbaum A., Hagel P., Sitsel O., Raisch T., Prumbaum D., .(2019). SPHIRE-crYOLO is a fast and accurate fully automated particle picker for cryo-EM. Commun Biol. 2(1): 218. 31240256 10.1038/s42003-019-0437-z PMC6584505

[18] Sun C., Gonzalez B. and Jiang W. (2022). Helical Indexing in Real Space. Sci Rep. 12(1): 8162. 35581231 10.1038/s41598-022-11382-7 PMC9114412

[19] Huber S. T., Kuhm T. and Sachse C. (2018). Automated tracing of helical assemblies from electron cryo-micrographs. J Struct Biol. 202(1): 1-12. 29191673 10.1016/j.jsb.2017.11.013 PMC5847486

[20] Punjani A., Rubinstein J. L., Fleet D. J. and Brubaker M. A. (2017). cryoSPARC: algorithms for rapid unsupervised cryo-EM structure determination. Nat Methods. 14(3): 290-296. 28165473 10.1038/nmeth.4169

[21] Sanchez J. C., Montemayor E. J., Ploscariu N. T., Parrell D., Baumgardt J. K., Yang J. E., Sibert B., Cai K. and Wright E. R. (2023). Atomic-level architecture of Caulobacter crescentus flagellar filaments provide evidence for multi-flagellin filament stabilization. bioRxiv.

[22] Iudin A., Korir P. K., Somasundharam S., Weyand S., Cattavitello C., Fonseca N., Salih O., Kleywegt G. J. and Patwardhan A. (2023). EMPIAR: the Electron Microscopy Public Image Archive. Nucleic Acids Res. 51(D1): D1503-D1511. 36440762 10.1093/nar/gkac1062 PMC9825465

[23] Kremer J. R., Mastronarde D. N. and McIntosh J. R. (1996). Computer Visualization of Three-Dimensional Image Data Using IMOD. J Struct Biol. 116(1): 71-76. 8742726 10.1006/jsbi.1996.0013

[24] Jumper J., Evans R., Pritzel A., Green T., Figurnov M., Ronneberger O., Tunyasuvunakool K., Bates R., Žídek A., Potapenko A., .(2021). Highly accurate protein structure prediction with AlphaFold. Nature. 596(7873): 583-589. 34265844 10.1038/s41586-021-03819-2 PMC8371605

[25] Pettersen E. F., Goddard T. D., Huang C. C., Meng E. C., Couch G. S., Croll T. I., Morris J. H. and Ferrin T. E. (2021). UCSF ChimeraX: Structure visualization for researchers, educators, and developers. Protein Sci. 30(1): 70-82. 32881101 10.1002/pro.3943 PMC7737788

[26] Emsley P., Lohkamp B., Scott W. G. and Cowtan K. (2010). Features and development of Coot. Acta Crystallogr. 66(4): 486-501. 10.1107/S0907444910007493 PMC2852313 20383002

[27] Liebschner D., Afonine P. V., Baker M. L., Bunkoczi G., Chen V. B., Croll T. I., Hintze B., Hung L. W., Jain S., McCoy A. J., .(2019). Macromolecular structure determination using X-rays, neutrons and electrons: recent developments in Phenix. Acta Crystallogr D Struct Biol. 10): 861-877. 31588918 10.1107/S2059798319011471 PMC6778852

[28] DiNunno N., Bianchini E. N., Liu H. and Wang J. C. (2023). Protein Structure Predictions, Atomic Model Building, and Validation Using a Cryo-EM Density Map from Hepatitis B Virus Spherical Subviral Particle. Bio Protoc. 13(14): e4751. 10.21769/BioProtoc.4751 PMC10367000 37497443

[29] Morin A., Eisenbraun B., Key J., Sanschagrin P. C., Timony M. A., Ottaviano M. and Sliz P. (2013). Collaboration gets the most out of software. eLife 2: e01456. 24040512 10.7554/eLife.01456 PMC3771563

